# A frame-shifted gene, which rescued its function by non-natural start codons and its application in constructing synthetic gene circuits

**DOI:** 10.1186/s13036-019-0151-x

**Published:** 2019-03-01

**Authors:** Kathakali Sarkar, Sayak Mukhopadhyay, Deepro Bonnerjee, Rajkamal Srivastava, Sangram Bagh

**Affiliations:** Biophysics and Structural Genomics Division, Saha Institute of Nuclear Physics, Homi Bhabha National Institute, Block A/F, Sector-I, Bidhannagar, Kolkata, 700064 India

**Keywords:** Frame-shifted gene, Non-natural start codons, Rescued function, Synthetic gene circuits, *E. coli*, Non-directed evolution of synthetic gene circuit

## Abstract

**Background:**

Frame-shifted genes results in non-functional peptides. Because of this complete loss of function, frame-shifted genes have never been used in constructing synthetic gene circuits.

**Results:**

Here we report that the function of gene circuits is rescued by a frame-shifted gene, which functions by translating from a non-natural start codon. We report a single nucleotide deletion mutation that developed in the λ-repressor cI within a synthetic genetic NOT gate in *Escherichia coli* during growth and through this mutation, a non-functional synthetic gene circuit became functional. This mutation resulted in a frame-shifted cI, which showed effective functionality among genetic NOT-gates in *Escherichia coli* with high regulatory ranges (> 300) and Hill coefficient (> 6.5). The cI worked over a large range of relative copy numbers between the frame-shifted gene and its target promoter. These properties make this frame-shifted gene an excellent candidate for building synthetic gene circuits. We hypothesized a new operating mechanism and showed evidence that frame-shifted cI was translated from non-natural start codon. We have engineered and tested a series of NOT gates made from a library of cI genes, each of which starts from a different codon within the first several amino acids of the frame-shifted cI. It is found that one form with start codon ACA, starting from the 3rd codon had similar repression behavior as the whole frame-shifted gene. We demonstrated synthetic genetic NAND and NOR logic-gates with frame-shifted cI. This is the first report of synthetic-gene-circuits made from a frame-shifted gene.

**Conclusions:**

This study inspires a new view on frame-shifted gene and may serve as a novel way of building and optimizing synthetic-gene-circuits. This work may also have significance in the understanding of non-directed evolution of synthetic genetic circuits.

**Electronic supplementary material:**

The online version of this article (10.1186/s13036-019-0151-x) contains supplementary material, which is available to authorized users.

## Background

The desired behavior of transcriptional synthetic gene circuits is a function of appropriate rate parameters, which are achieved by altering transcription and translational rates, strength of operating sites-transcription factor interaction and their relative copy numbers [1–3]. Transcription and translation rates are changed by altering the sequence of the promoter and ribosome binding sites (RBS) respectively, during iterative optimization of a gene circuit. The copy numbers are changed by changing the origin of replications in a plasmid [1, 2]. On the other hand, alteration of operating sites-transcription factor interaction by mutating a transcription factor, through directed evolution [4] is the gold standard approach for optimizing transcriptional gene circuits [5–7]. Directed evolution depends on multiple cycles of generating parallel and random mutations on promoter, RBS or genes, followed by proper screening to identify and isolate the best circuit behaviour. The mutation(s) in the gene alters the interaction parameters of the proteins with the other proteins and the DNA. This results change in kinetic parameters of a circuit. Mutations, which bring positive changes in the circuit behaviour are chosen for the next cycle. Mutations, which result negative changes in a circuit or loss of protein functions are no use and automatically discarded during the screening process [2, 4–7].

Single nucleotide deletion near the start codon results in a frame-shift, which in turn produces a non-functional peptide. Because of this complete loss of function, frame-shifted genes have never been used in constructing synthetic gene circuits.

Here we report a frame-shift mutated λ repressor cI gene, which rescued its function. We hypothesized a new mechanism and showed evidence that the frame-shifted gene might retain its function by getting translated from non-natural start codons. Here we define non-natural start codons as codons, which do not belong to known natural bacterial start codons, including rare ones. We successfully applied this frame-shifted cI in constructing synthetic genetic logic gates, which showed high Hill coefficient, dynamic ranges and workability range. This is also the first report of using a frame-shifted gene in constructing synthetic gene circuits.

## Results

### Functional behavior of frame-shifted λ repressor cI

**Fig. 1:**
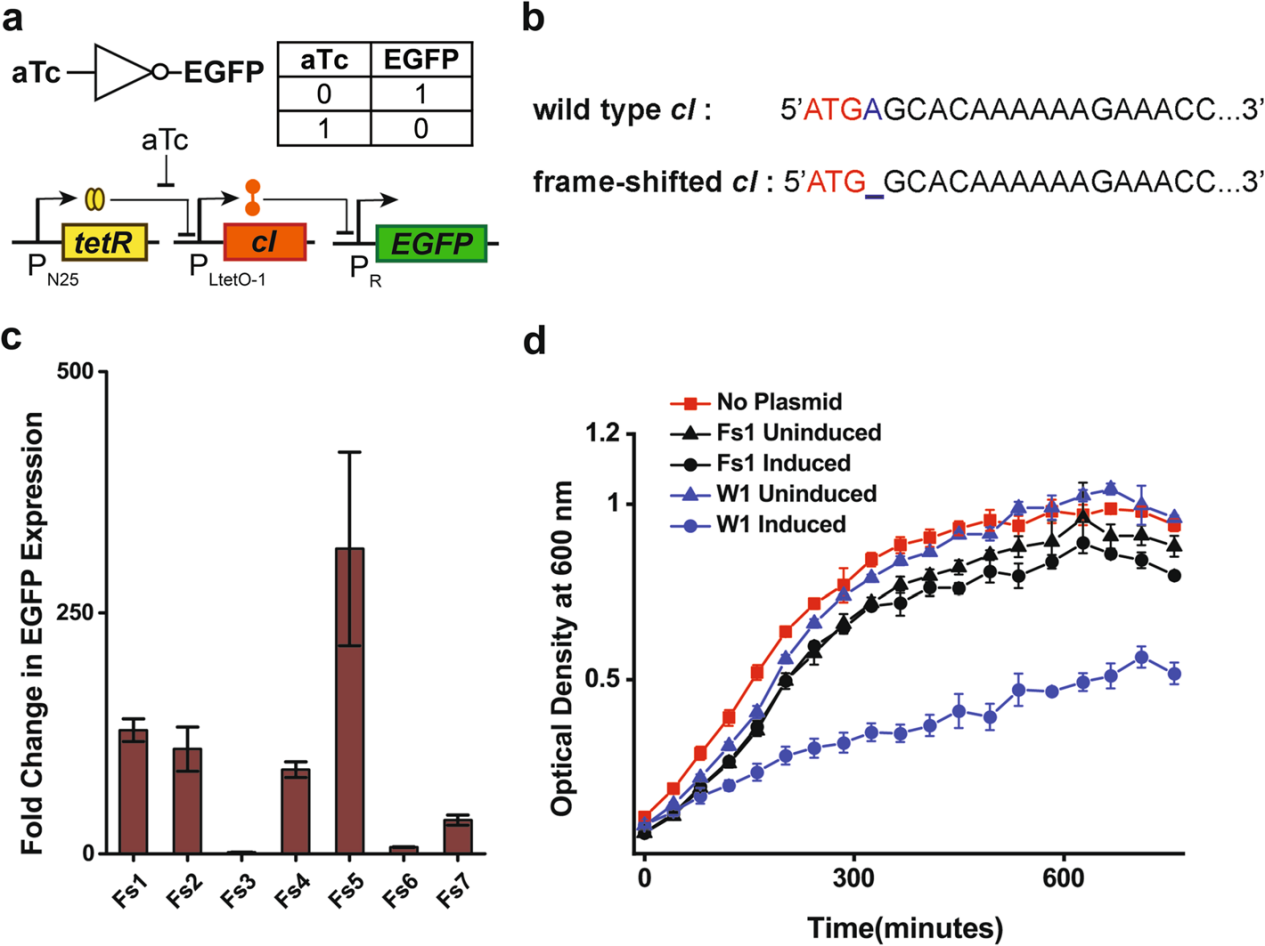
Frame-shifted cI and its function. **a**) Truth table and gene circuit design of the NOT gate. **b**) The partial sequence of wild type and single nucleotide deleted, frame-shifted cI. **c**) The fold changes in EGFP expression of the NOT gates between output logic level ‘1’ (high expression), when input is ‘0’ (absence of aTc) and output logic level 0 (low expression), when input is ‘1’ (200 ng/ml aTc, saturated concentration, see Fig. 2). The constructs Fs1–7 contain frame-shifted cI gene(Fs1: both frame-shifted *cI* and P_R_-*EGFP* in pUC, Fs2: frame-shifted *cI* in pUC and P_R_-*EGFP* in p15A, Fs3: frame-shifted *cI* in p15A and P_R_-*EGFP* in pUC, Fs4: both frame-shifted *cI* and P_R_-*EGFP* in ColE1, Fs5: frame-shifted *cI* in ColE1 and P_R_-*EGFP* in p15A, Fs6: frame-shifted *cI* in p15A and P_R_-*EGFP* in ColE1, Fs7: both frame-shifted *cI* and P_R_-*EGFP* in p15A). **d**) Comparison of growth curves of *E.coli* DH5αZ1 expressing wild type cI (W1, induced with 200 ng/ml aTc), frame-shifted cI (Fs1, induced with 200 ng/ml aTc) and other non-expressing states (0 ng/ml) that are W1 uninduced, Fs1 uninduced and without plasmid .W1 contains both wild type *cI* and P_R_-*EGFP* in pUC

The λ repressor cI, in its dimer form works as a strong repressor for the P_R_ promoter [8].During the process of making a genetic NOT gate W1 (Fig. 1a and Table 1), wild type cI gene was placed under P_LtetO-1_ promoter with a strong RBS [9] and enhanced green fluorescence protein (EGFP) gene was placed under P_R_ promoter with the same RBS, both in a high copy plasmid (pUCori, ~ 500 copies per cell [10]). After ligating the P_LtetO-1_-*cI* fragment in the vector containing P_R_ –*EGFP* cassette, 2 μl of the ligated product was heat transformed into chemically competent *E. coli* DH5αZ1 cells, which constitutively expresses lac repressor protein LacI and Tet repressor protein TetR from the bacterial genome [9]. The resultant TetR represses the P_LtetO-1_ promoters present in the plasmids [9]. Therefore, it was expected that in the absence of anhydrotetracycline (aTc), which allosterically binds to TetR proteins and frees the P_LtetO-1_ promoter for transcription [9], basal level expression of cI protein would be small and could not repress the P_R_ promoter. Thus EGFP must be expressed to give rise to green colonies. However, only a small fraction of green fluorescent colonies were observed, with the majority appearing white under blue transillumination.

**Table 1:** Various NOT gate constructs from wild type (W), frame-shifted (Fs) and truncated versions (T) of cI gene (*cI*)

Constructs	Nature of *cI*	Ori for P_LtetO-1_-*cI*	Ori for P_R_-*EGFP*
Fs1	frame-shifted *cI*	pUC	pUC
Fs2	frame-shifted *cI*	pUC	p15A
Fs3	frame-shifted *cI*	p15A	pUC
Fs4	frame-shifted *cI*	ColE1	ColE1
Fs5	frame-shifted *cI*	ColE1	p15A
Fs6	frame-shifted *cI*	p15A	ColE1
Fs7	frame-shifted *cI*	p15A	p15A
W1	wild type *cI*	pUC	pUC
W2	wild type *cI*	pUC	p15A
W3	wild type *cI*	p15A	pUC
W4	wild type *cI*	ColE1	ColE1
W5	wild type *cI*	ColE1	p15A
W6	wild type *cI*	p15A	ColE1
W7	wild type *cI*	p15A	p15A
T1	Truncated*cI*(T1^a^/*cI*-G, starting from glycine)	pUC	p15A
T2	Truncated*cI*(T2^a^/ *cI*-T3, starting from threonine 3)	pUC	p15A
T3	Truncated*cI*(T3^a^/ *cI*-K4, starting from lysine 4)	pUC	p15A
T4	Truncated*cI*(T4^a^/ *cI*-K5, starting from lysine 5)	pUC	p15A
T5	Truncated *cI*(T5^a^/ *cI*-K6, starting from lysine 6)	pUC	p15A
T6	Truncated*cI*(T6^a^/ *cI*-L8, starting from leucine 8)	pUC	p15A
T7	Truncated*cI*(T7^a^/ *cI*-T9, starting from threonine 9)	pUC	p15A
T8	Truncated*cI*(T8^a^/ *cI*-E11, starting from glutamate 11)	pUC	p15A
T9	Truncated*cI*(T9^a^/ *cI*-M41, starting from methionine 41)	pUC	p15A

^a^The non-natural start codons and their positions are shown in Fig. 3

The plasmids from both fluorescent and non-fluorescent colonies were extracted and sequenced. Results showed a single nucleotide deletion (SND) mutation at the 2^nd^codon of cI (Fig. 1b) in the plasmids from the green colonies, whereas white colonies showed the correct cI sequence. This SND resulted in a frame-shift in the cI gene (Fig. 1b). The deletion found in the cI gene from the green colonies was spontaneous. We did not find mutation in the PCR product of cI gene before ligation (Additional file 1: Figure S1).

To test the functional ability of this frame-shifted cI, if any, a series of 7 different NOT gate systems (Fs1-Fs7) were constructed, where the absolute as well as relative copy numbers of frame-shifted cI gene and P_R_ promoter-*EGFP* gene constructs were varied (Table 1). The constructs followed the general design as in Fig. 1a and were transformed into DH5αZ1 cells. Fluorescence from EGFP expression was measured in a multimode reader after growing the diluted cells from overnight culture in fresh Luria-Bertani (LB) medium with appropriate antibiotic for ~ 16 h at 37^o^ C without (0 ng/ml) and with (200 ng/ml) aTc, separately (See methods for details).

It was observed that frame-shifted cI effectively represses P_R_ promoter and resulted in reduced EGFP expression with a varying dynamic range changes (from1.5 times (Fs3) to as high as > 300 times (Fs5)) between 0 ng/ml aTc (input logic level ‘0’) and 200 ng/ml (input logic level ‘1’) aTc concentrations(Fig. 1c and Additional file 1: Figure S2a). Further, *E.coli* DH5αZ1 cells expressing frame-shifted cI (Fs1) showed higher growth rate, similar to cells without plasmids in comparison to the cells expressing wild type cI (Fig. 1d). Further, a scatter plot was made to estimate a simple relationship, if any, between the dynamic range and the ratio of approximate average copy numbers of the plasmid carrying frame-shifted cI and plasmid carrying P_R_-EGFP. No simple correlation was found (Additional file 1: Figure S3).

Next, the ultrasensitivity of 5 NOT gates with changes > 30 times (Fs1, Fs2, Fs4, Fs5 and Fs7) were measured as a function of aTc concentration and the experimental behavior was fitted with a Hill function:

1 $$ {\left[\mathrm{EGFP}\right]}_{\mathrm{ss}}=\mathrm{c}\left(\mathrm{b}+\frac{1}{1+\kern0.5em {\left(\frac{\left[\mathrm{aTc}\right]}{\mathrm{K}}\right)}^{\mathrm{n}}}\right) $$

Where [EGFP]_ss_ represents the normalized fluorescence from EGFP expression, ‘b’ is the basal level fluorescence, [aTc] is the concentration of aTc in the media, ‘n’ is the Hill coefficient, ‘K’ is the Hill constant and ‘c’ is the scaling factor. The Hill coefficient ‘n’is a measure of the ultrasensitivity of a synthetic gene circuit.

**Fig. 2:**
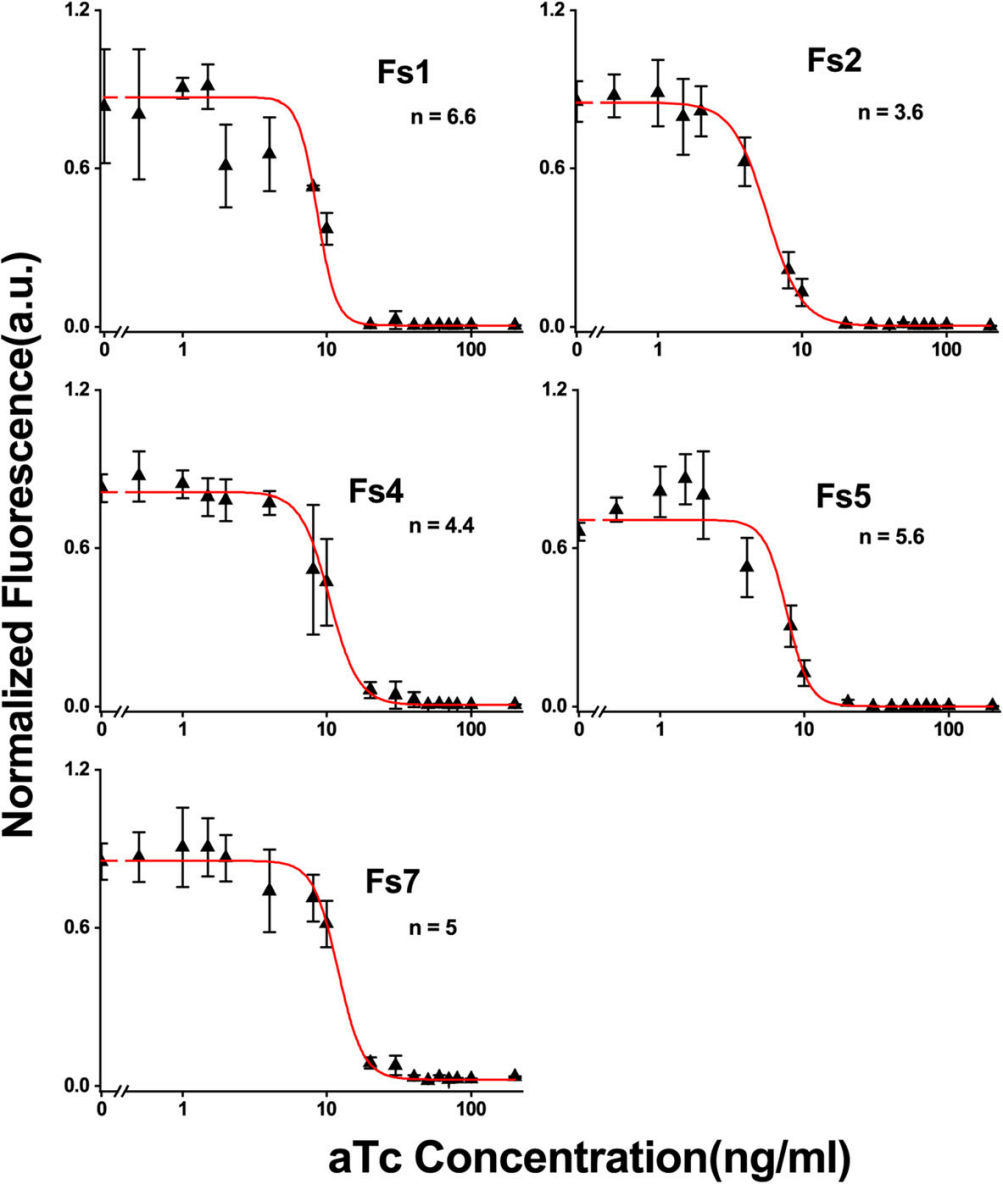
Ultrasensitivity of NOT gates with frame-shifted cI. Normalized fluorescence from EGFP expression of various NOT gate constructs using frame-shifted cI as a function of various aTc concentrations. The data were fitted (solid line) with a Hill function (Eq. (1)). The Hill coefficient ‘n’ values were shown corresponding to each NOT gate constructs

Figure 2 shows experimental results along with Hill coefficient (n) values from the fitting. Other parameters are shown in Additional file 1: Table S1. A sharp transition in EGFP expression between low and high aTc concentration with a Hill coefficient (3.6–6.6) was observed. In contrast to the frame-shifted cI constructs (Fs1-Fs7), the similar constructs with wild type cI (W1-W7) were fully repressed under all conditions (Additional file 1: Figure S2b). Even a basal level leakage of wild type cI from fully repressed P_LtetO-1_ promoters (without adding any aTc in the media) in low copy plasmids (p15A ori, approximately 20–30 copies per cell [9] in DH5αZ1) was enough to repress EGFP expression from P_R_ promoters present in high copy plasmids (W3) (pUCori) (Additional file 1: Figure S2b). This experimental observation was also partially supported by thermodynamic calculations using an RBS calculator [11, 12], which showed that for the same RBS, the cI gene has ~ 1170 times higher translation rate than EGFP (Additional file 1: Table S2). This higher translation rate might produce small but sufficient numbers of cI proteins from the fully repressed P_LtetO-1_ promoterto strongly repress the P_R_ promoter.

### Hypothesis and evidence of non-natural start codons

Next, the sequence of the frame-shifted cI was analyzed using a bioinformatics tool (ExPASy Translate, SIB Swiss Institute of Bioinformatics). The first base ‘A’ in the second codon of the gene was deleted, resulting in a frame-shift mutation. The resultant protein sequence was completely different from the wild type cI and thus must not have any functional similarity with the wild type cI (Additional file 1: Figure S4a). However, a continuous peptide sequence after 40 amino acids with normal start codon AUG (ATG in gene) was found in frame until the normal stop codon (Additional file 1: Figure S4a). A NOT gate construct (T9) with this truncated version was made and tested. In experiment, this truncated version showed only a 2-fold reduction in EGFP expression from the P_R_ promoter and thus could not explain the high degree of repression (Additional file 1: Figure S4b).

**Fig. 3:**
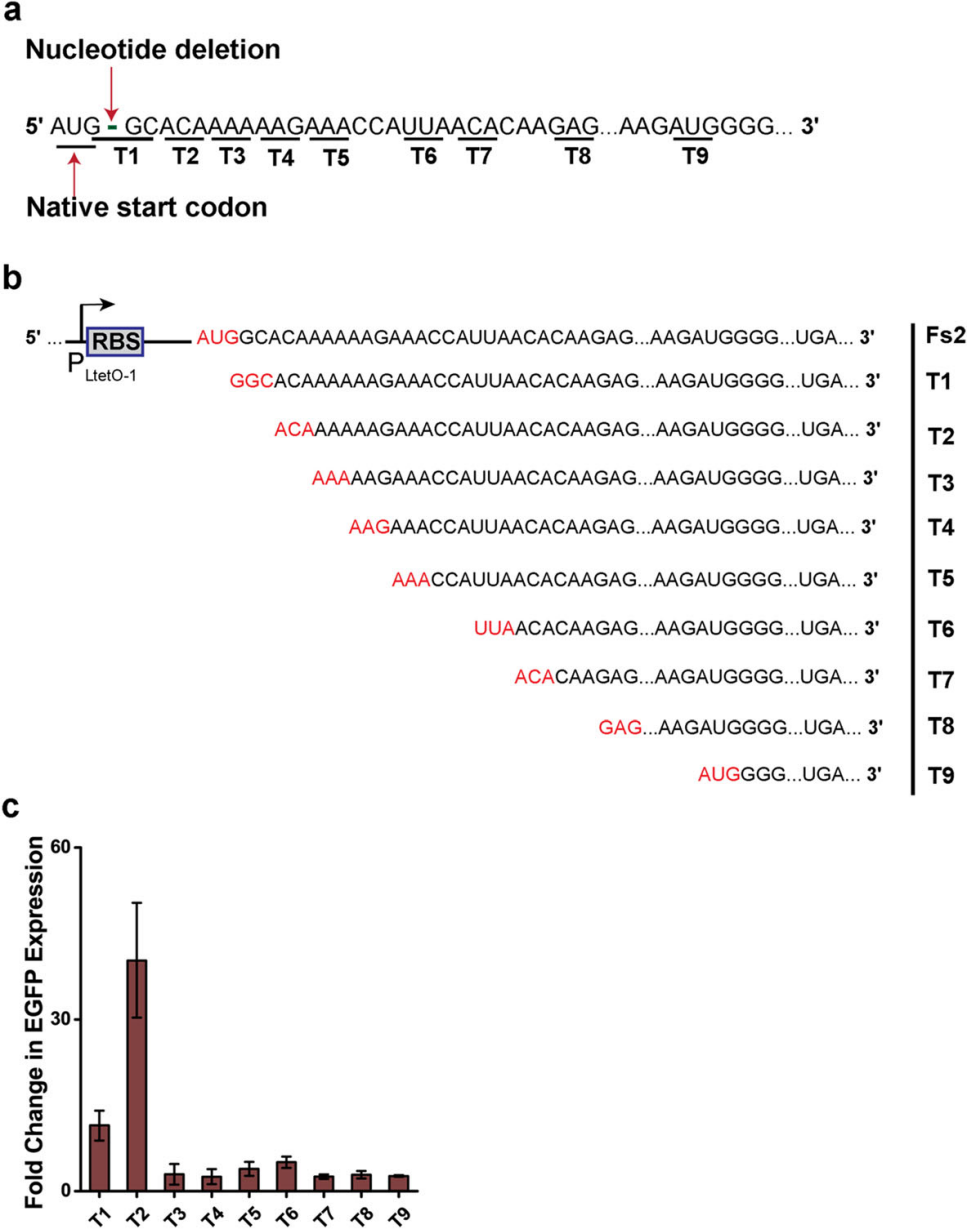
**a**) Postulated truncated cI with non-natural start codons (T1-T8). T9 is the AUG start codon after 40th codon. **b**) Frame-shifted cI in Fs2 NOT gate construct is replaced by individual truncated cIs (T1-T9) without changing the P_LtetO-1_ promoter, native RBS and the linker region between RBS and ORF, and co-transformed with P_R_-EGFP carrying plasmid (p15A Ori) to create 9 NOT gates. The start codons of frame-shifted cI and truncated cIs are in red and the stop codons (UGA) are also shown. **c**) The fold change in EGFP expression of the NOT gates with various truncated cIs, T1-T9 between output ‘ON’ (input OFF; 0 ng/ml aTc) and ‘OFF’ states (input ON; 200 ng/ml aTc)

Next, we hypothesized that instead of AUG, the frame-shifted cI might translate from any other codon (non-natural start codons) and bring the protein in frame until the natural stop codon of the wild type cI. We found 8 N-terminus truncated versions of the cI in frame (Fig. 3a) within first 11 codons for consideration.

To test our hypothesis, a library of plausible (total 8) truncated genes within the first 11 codons with associated non-natural start codons (Fig. 3a) was made (T1-T8 in Table 1). Eight separate constructs were made by replacing frame-shifted cI gene with the truncated genes (T1-T8) in NOT gate construct Fs2 (Table 1, Fig. 3b and Additional file 1: Figure S5d), where frameshifted cI was under P_LtetO-1_ promoter followed by the RBS and a linker of an optimal length for translational initiation [13, 14]. The new plasmids (T1-T8) were co-transformed with the plasmid carrying P_R_-EGFP with p15A Ori same as in Fs2 NOT gate construct (Table 1).

One NOT gate with truncated construct (T2) showed 40-fold repression in EGFP expression from the P_R_ promoter (Fig. 3c), which matches the repression value (~ 34 times) of Fs7.In T2, the truncated cI was translated from 3rd codon ACA and had the first two amino acids (Met and Ser) truncated from the N-terminus. Other truncated constructs with other non-natural start codons (Fig. 3a and Fig. 3b) showed smaller repression (~ 2–11-fold; Fig. 3c and Additional file 1: Figure S6).

### Synthetic genetic logic gates with frame-shifted cI

**Fig. 4:**
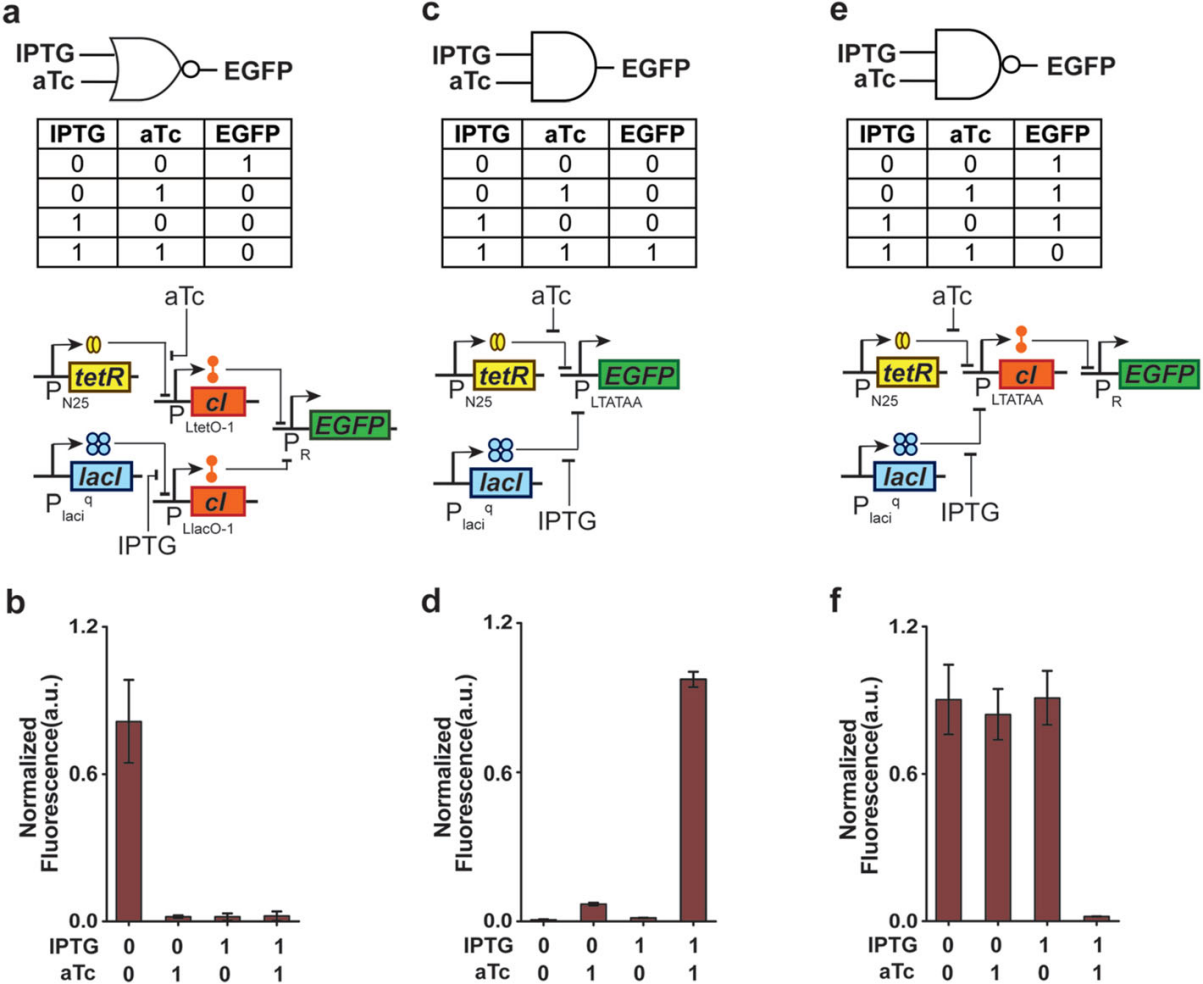
Logic operations with frame-shifted λ repressor. Synthetic NOR and NAND gates are constructed with frame-shifted cI. The truth table and biochemical designs of **a**) NOR, **c**) AND and **e**) NAND logic gates. Experimental behaviour with logic levels for **b**) NOR, **d**) AND and **f**) NAND gate is also shown. Each logic gate has two inputs: IPTG and aTc and one output EGFP. The “0” and “1” inputs are basically particular concentrations of both IPTG and aTc (0 mM IPTG for IPTG “0” input, 0 ng/ml aTc for aTc “0” input, 10 mM IPTG for IPTG “1” input and 200 ng/ml aTc for aTc “1” input)

Next, to demonstrate the functional potential of frame-shifted cI, synthetic genetic universal NOR and NAND logic gates with frame-shifted cI were designed and constructed (Fig. 4). Experimental results showed the NOR logic behavior (Fig. 4b), where only in the absence of both input signals IPTG and aTc (input logic level: 0,0), the output EGFP showed high expression (output logic: 1).

The NAND logic gate was designed as an AND gate followed by a NOT gate. First we made a minimal AND gate by designing, constructing and testing a hybrid synthetic promoter P_LTATAA_. This hybrid promoter can be repressed by both LacI and TetR proteins (Fig. 4c) and thus always becomes repressed in DH5αZ1 cell and can only be activated in the simultaneous presence of aTc and Isopropyl β-D-1-thiogalactopyranoside (IPTG), which allosterically binds to LacI and frees the operator sites in the promoter. The design of this hybrid promoter was based on a previous design [15] but used different distances between operator sites, positions and numbers of transcription factor (TF) binding sites (Additional file 1: Table S3). EGFP was placed under this promoter and its successful AND logic behavior was tested (Fig. 4d).

Next, to make a NAND logic system (Fig. 4e), the output gene EGFP of this AND gate was replaced with the frame-shifted cI, placed in a medium copy plasmid (ColE1 ori) and co-transformed with P_R_-*EGFP* construct (p15A ori) into DH5αZ1 cells (Fig. 4e). In this full NAND system, the output of the AND gate (frame-shifted cI) was connected with a NOT gate (P_R_ promoter) and its output EGFP. Figure 4f showed the NAND logic behavior, where the output signal is off only when both input signals are present. The difference between OFF and ON states for both NOR and NAND gate were ~ 40 times.

## Discussion

The λ repressor cI with P_R_ promoter has been used for constructing several gene circuits [7, 16, 17]. However, the strength of the associated RBS generally needs to be reduced [7]. We showed that even a basal level leakage of wild type cI from fully repressed pLtetO-1 promoter was enough to repress the P_R_ promoter completely. Thus NOT gates made from wild type cI were nonfunctional. However, the function of the NOT gates were rescued when the frame shifted cI was used. The NOT gates constructs created in this study showed high fold change, ultrasensitivity (large Hill coefficient) and normal growth rate as per the best transcriptional NOT gates [18] reported. However, a promoter- repressor pair in a gene circuit may not work effectively in other circuits if there is a mismatch in their relative copy numbers [1]. Similarly, the dynamic range of a specific circuit may not match with the downstream promoter-gene pairs within a larger circuit [1]. In those cases, tedious transcriptional and/or translational optimization is often required to match the [18–21] cellular availability of a repressor genes with respect to the promoters. Here, we have shown that, frame-shifted cI can work over a large range of plasmid copy numbers, as evident from the Fig. 2, which shows successful repression behavior even when the relative numbers of plasmid copy separately carrying frame-shifted cI and its target promoter are varied in all possible ways (see Table 1). This is one of the most attractive properties of this frame-shifted cI, which most of the other known transcription factor-promoter pairs lack. We think this property of frame-shifted cI originates from the inefficient translation from the non-natural start codons. The inefficient translation would not allow the (truncated) cI protein to reach the saturated level even when we used high copy number plasmid (please see below for more details). Synthetic genetic NOR and NAND gates with frame-shifted cI were successfully demonstrated. The ultrasensitivity observed in those logic circuits is as per the standard transcriptional logic gates reported [22]. It isimportant to note that, in all synthetic gene circuits described here, frame-shifted cI were used with strong promoter and RBS unlike wild type cI found in the literature [7].

This single nucleotide deleted, frame-shifted λ repressor cI was produced spontaneously during growth of *E. coli* carrying wild type cI. We hypothesized and showed evidence that frame-shifted cI became functional by producing truncated version of cI translated from non-natural start codons. It suggests a new mechanism of rescued functionality from a frame-shifted gene. It was previously observed that cI proteins with inserted random linkers in early positions of N-terminal regions showed high to low repression ability against P_L_ promoter [23, 24]. However, no studies were performed by replacing or removing AUG (ATG) start codon. The start codons used in our study do not belong to the other lesser used and rare but known start codons in bacteria like GUG, UUG, CUG, AUU, AUC and AUA [14, 25]. Thus we defined them as non-natural start codons. A recent study [14] showed that translation with varied rate can be achieved with various non natural start codons in super folder green fluorescence protein (sfGFP), which was placed under T7 promoter in *E.coli* and ACA start codon resulted in a decrease of ~ 1200 (in rich defined media) and ~ 5000 (in LB media) in sfGFP expression compared to the AUG. Further, the position of this non-natural start codon in frame-shifted cI was 11 nucleotides away from the RBS, which was within the limit for good translation initiation rate in bacteria [13]. This suggested that the frame-shifted cI resulted in functional truncated cI, translating from non-natural ACA codon and its number in the *E. coli* was reduced proportionally in comparison to wild type cI to give repression with good dynamic range. This was also supported by the observation that we found higher cellular growth rate with frame-shifted cI than wild type cI (Fig. 1d). We reasoned that low amount of truncated but functional cI did not exert high metabolic load, thus it has negligible effect on growth rate.

In our study, we had two interesting observations. First the mutation gave an adaptive advantage as evident from the fact that in induced condition, the plasmids carrying frame-shifted cI had higher growth rate than the plasmid carrying wild type cI. Second, this is an example, where non-directed mutation makes a non-functional gene circuit into a functional one.

## Conclusion

Taken together, our work suggests a new view on a frame-shifted gene in the light of non-natural start codons and inspires future exploration of using frame-shifted genes and non-natural start codons as a new axis for building and optimizing gene expression in synthetic genetic circuits. Further, as this frame-shifted cI requires almost no alteration in its transcription and translation rateswithin a large range of plasmid copy numbers, it could be a better alternative to wild type cI for constructing synthetic gene circuits. This study raises interesting questions about non-directed evolution of synthetic genetic circuits.

## Methods

### Promoters, primers, genes, plasmids and bacterial cell strains

Three base plasmids pTA1EGFP, pTA2EGFP and pTC3EGFP were prepared (Additional file 1: Table S4, Additional file 1: Figure S5). EGFP gene, P_LtetO-1_ promoter, ColE1 and p15A origin of replication (ori), ampicillin and chloramphenicol resistance genes were taken from plasmids: pOR-EGFP-12 and pOR-Luc-31 (gift from Prof. David McMillen, University of Toronto, Toronto, Canada). The pUCori was PCRed from pmCherry-N1 (Clontech) using Primer_34 and Primer_35 (all the primers used in this study was listed in Additional file 1: Table S5) and incorporated into pTA2EGFP to get pTA1EGFP. All primers were synthesized from Integrated DNA Technologies, Singapore. P_R_ promoter along with RBSwas constructed by self-priming PCR of two primers Primer_9 and Primer_10. EGFP gene was fused to this promoter-RBS fragment by a fusion PCR with Primer_8 and Primer_11. This ultimately led to full-length P_R_-EGFP construct. This P_R_-EGFP construct was flanked by *Xho*I and *Xba*I and it included the same RBS as that in pTA2EGFP. Hybrid promoter for AND gate, P_LTATAA_ had two TetR binding sites and three LacI binding sites. At first, one DNA fragment containing − 10 and − 35 hexamers, transcription start site, two TetR binding sites and two LacI binding sites was synthesized by Invitrogen GeneArt Gene Synthesis service, Thermo Fischer. Next we incorporated additional LacI binding site into that synthesized DNA fragment to get full length P_LTATAA_ flanked by *Xho*I and *Eco*RI sites using Primer_12 and Primer_13. The NAND gate was dependent on this hybrid promoter. Once all the additional promoters were constructed, P_LtetO-1_ was replaced by P_R_, P_LlacO-1_ or P_LTATAA_ in the base plasmids as required to generate others.

The λ repressor cI gene was PCRed from *E. coli* K-12 (procured from IMTECH, Chandigarh, India; MTCC number 1302) genomic DNA with appropriate primers (Primer_1-Primer_4) and its truncated variants were PCRed with appropriate primers using wild type cI gene in plasmid as the template. Those were cloned into base plasmids between *Kpn*I and *Xba*I under P_LtetO-1_, P_LlacO-1_ or P_LTATAA_ as appropriate (Additional file 1: Figure S5). Frame-shifted cI (cI single nucleotide deletion (SND) mutant) was generated spontaneously during growth. For different constructs, frame-shifted cI was restriction digested and inserted in appropriate plasmidswith varying copy numbers. A DNA fragment containing *Bam*HI and *Pst*I sites was synthesized and placed between *Aat*II and *Xho*I in order to introduce two genetic parts within a single plasmid (pLA1SEGFP and PRA1SEGFP)(Additional file 1: Figure S5). We also changed different parts of pLA1SEGFP and PRA1SEGFP to get series of plasmids having various combinations of replication origins (pUC, ColE1 and p15A), antibiotic resistance genes (*Ap*^r^ and *Cm*^r^), promoters (P_LtetO-1_, P_LlacO-1_, P_R_ and P_LTATAA_) and genes of interest (EGFP, wild type cI and cI SND mutant) (Additional file 1: Figure S5). We PCRed the second genetic cassette with the primers having *Pst*I and *Bam*HI sites in their flanking regions (Primer_36-Primer_39) for successful incorporation between *Bam*HI and *Pst*I in plasmids. The second genetic cassette incorporated within the DNA fragment was always placed upstream of the first cassette in the opposite direction. All PCR reactions were carried out by either KOD Hot Start DNA polymerase (Merck Millipore), Pfu Turbo Hotstart PCR Master Mix (Agilent Technologies) or Phusion High-Fidelity PCR Master Mix with HF Buffer (New England BioLabs). Plasmids were transformed into chemically competent *E. coli* cells. Cloning and plasmid amplification was performed in DH5α strain and all experiments were carried out in DH5αZ1 cells. Transformed cells were grown in LB-Agar, Miller (Difco, Beckton Dickinson) plates with appropriate antibiotics, followed by overnight liquid culture in LB broth from single colonies at 37 °C with appropriate antibiotics. Plasmids were extracted and sent for sequencing. Plasmids with the correct sequences were used for further experiments. All restriction enzymes and T4 DNA ligase were bought from New England BioLabs; plasmid isolation, gel extraction and PCR purification kits from QIAGEN; ampicillin and chloramphenicol from Himedia; kanamycin, aTc and IPTG from Sigma Aldrich and Abcam. Final concentration of the antibiotics in the growth media was 100 μg/ml, 34 μg/ml and 50 μg/ml for ampicillin, chloramphenicol and kanamycin respectively. Sequencing of plasmids, genes and promoters were performed by Eurofins Genomics India Pvt. Ltd., Bangalore, India.

### Cell growth for genetic constructs characterization

Cells were grown overnight in LB-liquid media with antibiotics, re-diluted 100 times in fresh LB media with antibiotics and inducers IPTG or aTc as appropriate and again grown for 16 h at 37^o^ C, ~ 250 rpm. Cells were washed twice and re-suspended in phosphate buffered saline (PBS, pH 7.4) for taking fluorescence measurements. 0 ng/ml of aTc (OFF state in input) and 200 ng/ml aTc (ON state in input) were used for determining ON and OFF states in fluorescence output. For dose response experiments with aTc, 0 ng/ml, 200 ng/ml and 15 more intermediate aTc concentrations (0.5 ng/ml, 1 ng/ml, 1.5 ng/ml, 2 ng/ml, 4 ng/ml, 8 ng/ml, 10 ng/ml, 20 ng/ml, 30 ng/ml, 40 ng/ml, 50 ng/ml, 60 ng/ml, 70 ng/ml, 80 ng/ml and 100 ng/ml) were used. IPTG were used as 0 mM (for OFF state in input) and 10 mM (for ON state in input) during the characterization of the AND, NOR and NAND gates.

### Fluorescence and optical density (OD) measurements

Cells were diluted in PBS (pH 7.4) to reach around OD_600_ 0.8, loaded onto 96-well multiwell plate (black, Greiner Bio-One) for measurement using Synergy HTX Multi-Mode reader (Biotek Instruments, USA). For fluorescencemeasurements, the cells were excited by a white light source that had been passed through an excitation filter 485/20 nm and emission was collected by 516/20 nm bandpass filter with appropriate gain. The OD_600_ was also measured in the same instrument. The raw fluorescence values were divided by respective OD_600_ values and thus normalized to the number cells. Auto-fluorescence was measured as average normalized fluorescence of the untransformed DH5αZ1 set (no plasmidset) and subtracted from the normalized fluorescence value of the experimental set. Data was been taken for at least 3 biological replicates for each condition.

### Data analysis and fitting

All data analysis and fitting was performed in OriginPro 2015 (OriginLab Corporation, USA). The dose response curves (Fig. 2) were fitted with the Hill equation (Eq. (1)) using built in Levenberg Marquardt algorithm, a damped least squares (DLS) method.

### Calculating translational rate in RBS calculator [11, 12]

Translation initiation rate for EGFP and wild type cI was calculated from “evaluate RBS library” design methods in RBS Library Calculator v2.0, (provided by Prof. Haward Salis, Penn State University, Pennsylvania, USA) considering *Eco*RI site and its upstream P_LletO-1_ promoter sequence as the pre-sequence, either *EGFP* or wild type *cI* as the protein coding sequence, RBS present in base plasmids along with conserved linker GGTACC (*Kpn*I site) as degenerate RBS sequence and *E. coli* str. K-12 substr. MG1655 as the organism.

### Bioinformatics tool

Analysis of the sequence for wild type cI and cI SND mutant was carried out in ExPASy Translate, SIB Swiss Institute of Bioinformatics.

## Additional file

Additional file 1: **Figure S1.** Sequencing results for PCRed *cI* gene. **Figure S2** Characterization of wild type (WT) cI and frame-shifted cI. **Figure S3** Correlation between the dynamic range and the approximate ratio of plasmid copy number carrying frame-shifted cI and P_R_-*EGFP* construct. **Figure S4** Open reading frames of wild type cI, frame-shifted cI and truncated cI started from amino acid M41. **Figure S5** Generic plasmid maps constructed in this study. **Figure S6** Characterization of the NOT gates repression behaviour with postulated truncated variants of cI. **Table S1** Curve fitting parameter values. **Table S2** Translation initiation rates for EGFP and cI calculated from RBS calculator. **Table S3** List of promoters. **Table S4** List of plasmids. **Table S5** List of primers. (DOCX 4535 kb)
