# Supplementary material for: A frame-shifted gene, which rescued its function by non-natural start codons and its application in constructing synthetic gene circuits
Source: J Biol Eng. 2019 Mar 1;13:20. doi: 10.1186/s13036-019-0151-x (PMC6397469; doi:10.1186/s13036-019-0151-x)
Supplement: Supplementary file 1 — Figure S1. Sequencing results for PCRed cI gene. Figure S2 Characterization of wild type (WT) cI and frame-shifted cI. Figure S3 Correlation between the dynamic range and the approximate ratio of plasmid copy number carrying frame-shifted cI and PR-EGFP construct. Figure S4 Open reading frames of wild type cI, frame-shifted cI and truncated cI started from amino acid M41. Figure S5 Generic plasmid maps constructed in this study. Figure S6 Characterization of the NOT gates repression behaviour with postulated truncated variants of cI. Table S1 Curve fitting parameter values. Table S2 Translation initiation rates for EGFP and cI calculated from RBS calculator. Table S3 List of promoters. Table S4 List of plasmids. Table S5 List of primers. (DOCX 4535 kb) [file 13036_2019_151_MOESM1_ESM.docx]

Supporting Information

**A frame-shifted gene, which rescued its function by non-natural start codons and its application in constructing synthetic gene circuits**

Kathakali Sarkar, Sayak Mukhopadhyay, Deepro Bonnerjee, Rajkamal Srivastava and Sangram Bagh *

*Address for correspondence

Biophysics and Structural Genomics Division

Saha Institute of Nuclear Physics, Homi Bhaba National Institute

Block A/F, Sector-I, Bidhannagar, Kolkata 700064 INDIA

E-mail: sangram.bagh@saha.ac.in


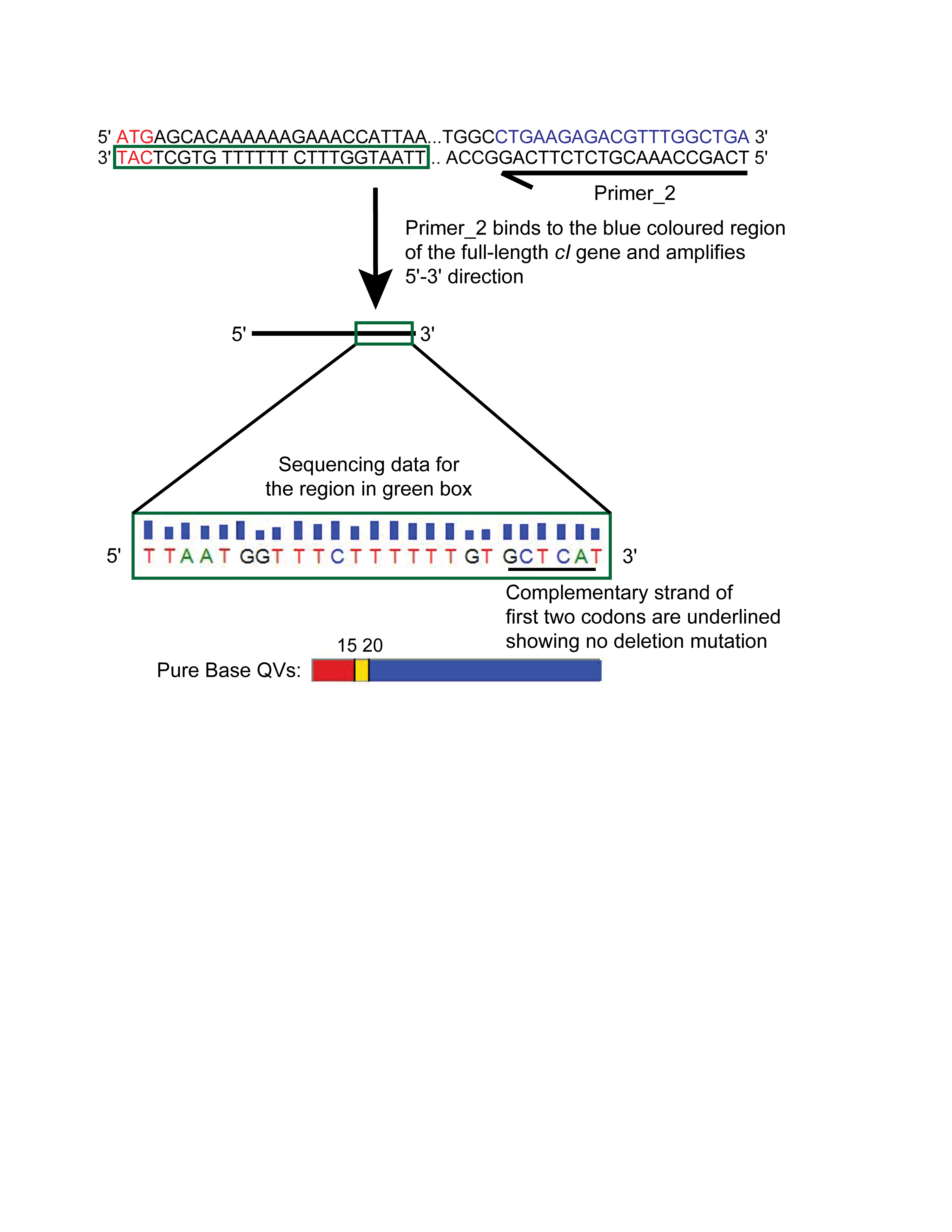


**Figure S1**. **Sequencing results for PCRed *cI* gene.** The region (green box) near start codon (shown in red in the top panel) was sequenced through a reverse primer Primer_2 (Supplementary Table S5), which primes at 69 bases downstream from the start codon. Primer_2 gives the sequencing data for the complementary strand (bottom strand). Sequencing data is showed in the bottom panel. The blue bar denotes the quality of the sequencing at that base. The quality of the data can be estimated from the color scale bar for quality value (QV) score per base. Quality value, also known as confidence score defines the chance of a base call to be wrong during DNA sequencing. For example, QV = 20 suggests a 10^-2^ chance of an incorrect base call. QV 0 to 14 (shown in red) represents poor quality data, QV 15 to 20 (shown in yellow) suggests nearly correct data and QV more than 20 (shown in blue) indicates good quality data.


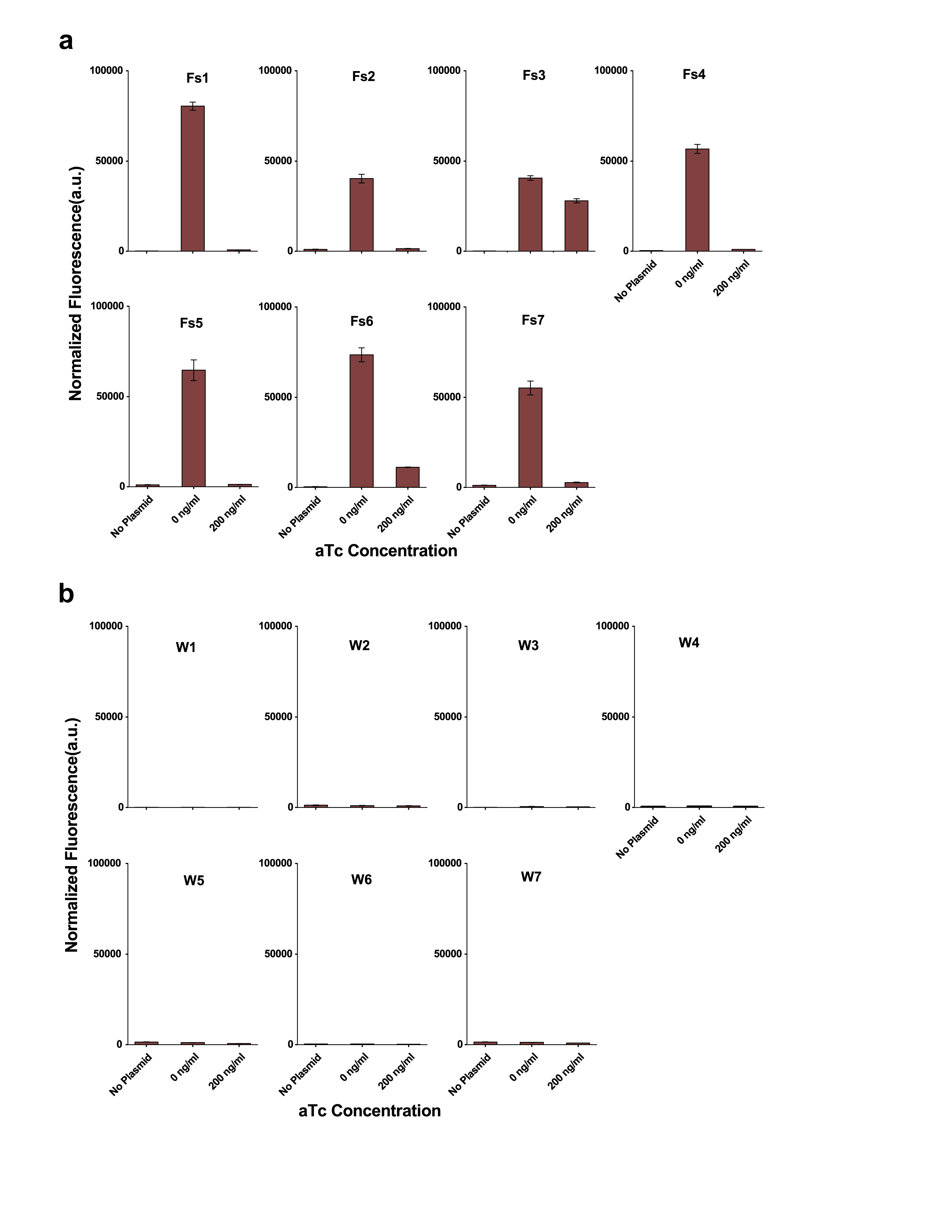


**Figure S2**. **Characterization of wild type (WT) cI and frame-shifted cI.** (a) The output behavior (normalized fluorescence from EGFP expression) of various NOT gates (Fs1-Fs7) as a function of input signals (0 ng/ml aTc, "0"or "OFF" state) and (200 ng/ml, "1"or "ON" state). The figures also include the normalized auto-fluorescence from the *E. coli* DH5αZ1 cells without plasmids for comparison. (b) The behavior of wild type cI in similar constructs (W1-W7). The fluorescence values are similar to auto-fluorescence, suggesting complete repression of the EGFP expression from P_LtetO-1_ promoter.


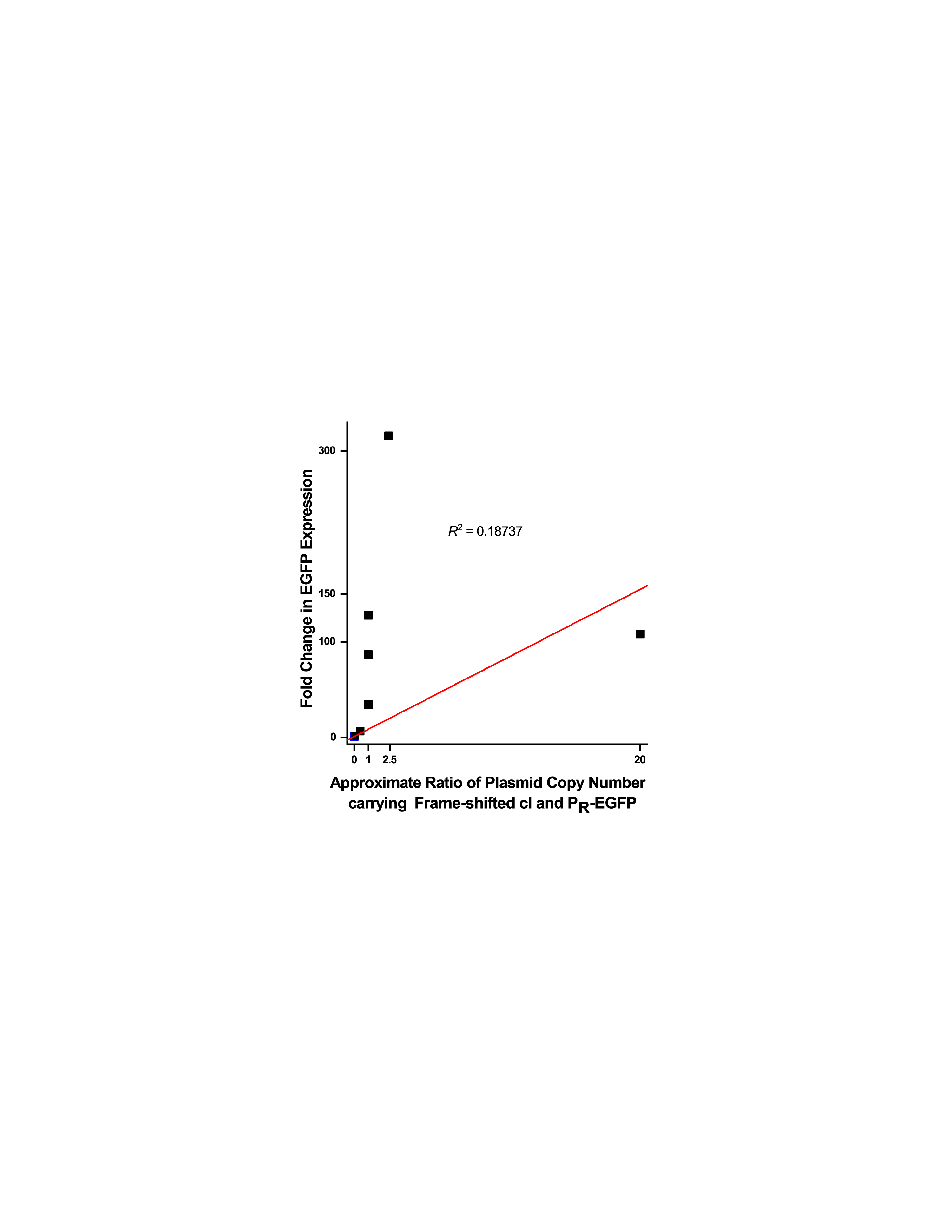


**Figure S3**. **Correlation between the dynamic range and the approximate ratio of plasmid copy number carrying frame-shifted cI and P_R_-*EGFP* construct**. The scatter plot was made by fixing the intercept at (0,1). The point colored in blue, representing the situation when there is no frame-shifted cI in the system and hence the fold change in EGFP expression is 1. Approximate average copy number for plasmids having origin pUC, ColE1 and p15A was taken as 500 (1), 60 (2) and 25 (2) respectively.


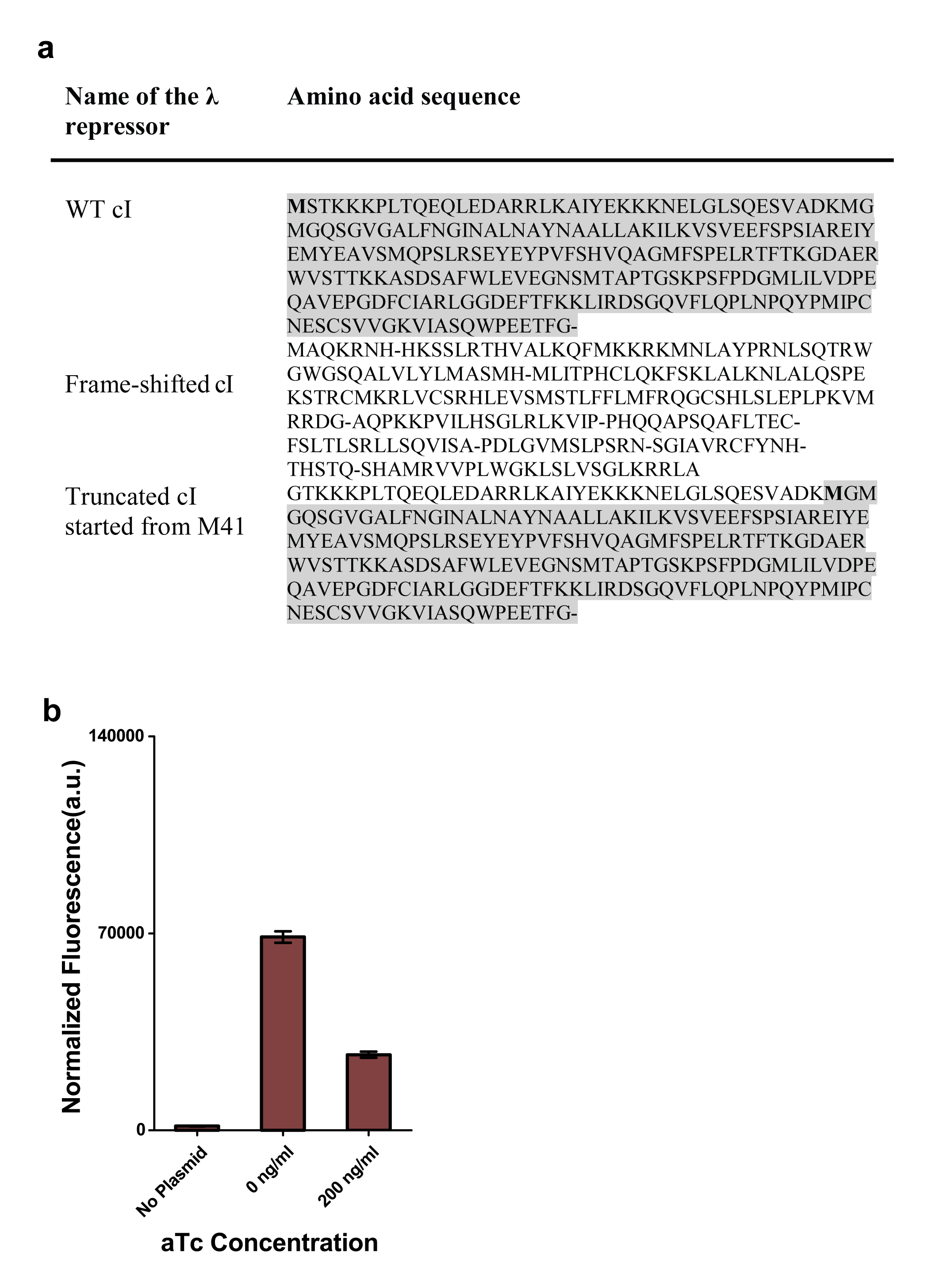


**Figure S4**. **Open reading frames of wild type cI, frame-shifted cI and truncated cI started from amino acid M41.** (a) Open reading frames for wild type cI (WT cI), frame-shifted cI and truncated cI starting after 40^th^ amino acid (construct T9 in Table 1 in main text) with a start codon ATG were obtained from ExPASy Translate tool. Amino acid methionine coded by the ATG start codon (M) is shown in bold. ORFs similar to the wild type cI are highlighted in grey. "-" represents the presence of stop codon in mRNA sequence. ORF for frame-shifted cI shows the frame shift and additional stop codons after deletion mutation. (b) The repression behavior of the truncated cI T9 (See Table 1 in main text) in *E. coli.* The truncated version of cI was constructed and tested with 0 ng/ml and 200 ng/ml aTc.


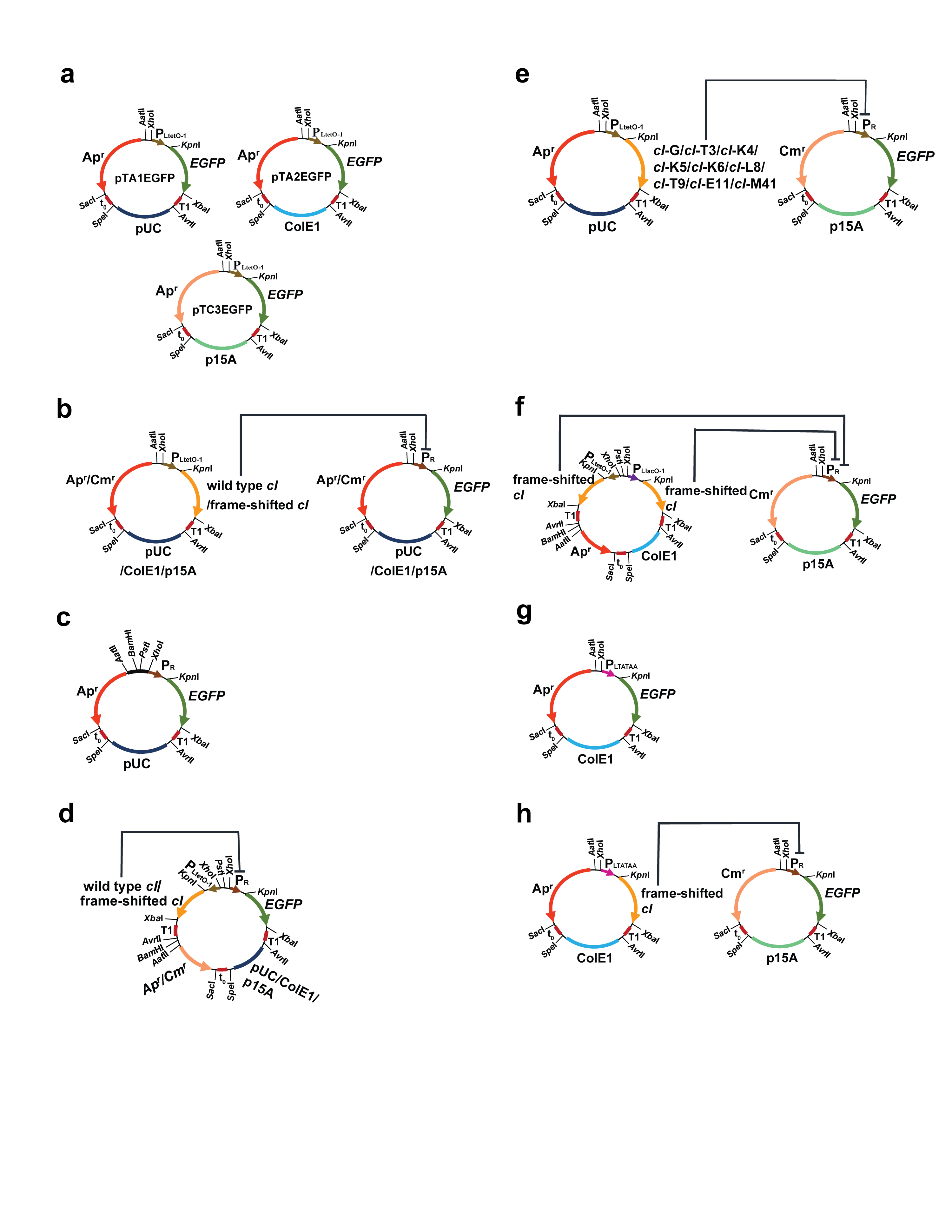


**Figure S5**. **Generic plasmid maps constructed in this study.** (a) Three base plasmids pTA1EGFP, pTA2EGFP and pTC3EGFP. (b) General plasmid model for P_LtetO-1_-*cI* and P_R_-*EGFP* NOT gate where *cI* and *EGFP* are under the control of origins of replication (ori) from two different incompatibility groups. (c) Base plasmid for incorporating additional gene cassette between *Bam*HI and *Pst*I, upstream of the existing gene cassette. (d) Model plasmid for *cI*-*EGFP* NOT gate constructs where both *cI* and *EGFP* are under the control of same ori. (e) Design for truncated *cI*-*EGFP* constructs where truncated λ repressor genes are under the control of pUC ori and *EGFP* is under the regulation of p15A ori. Plasmid constructs for (f) the NOR gate, (g) the AND gate and (h) the NAND gate are also represented.


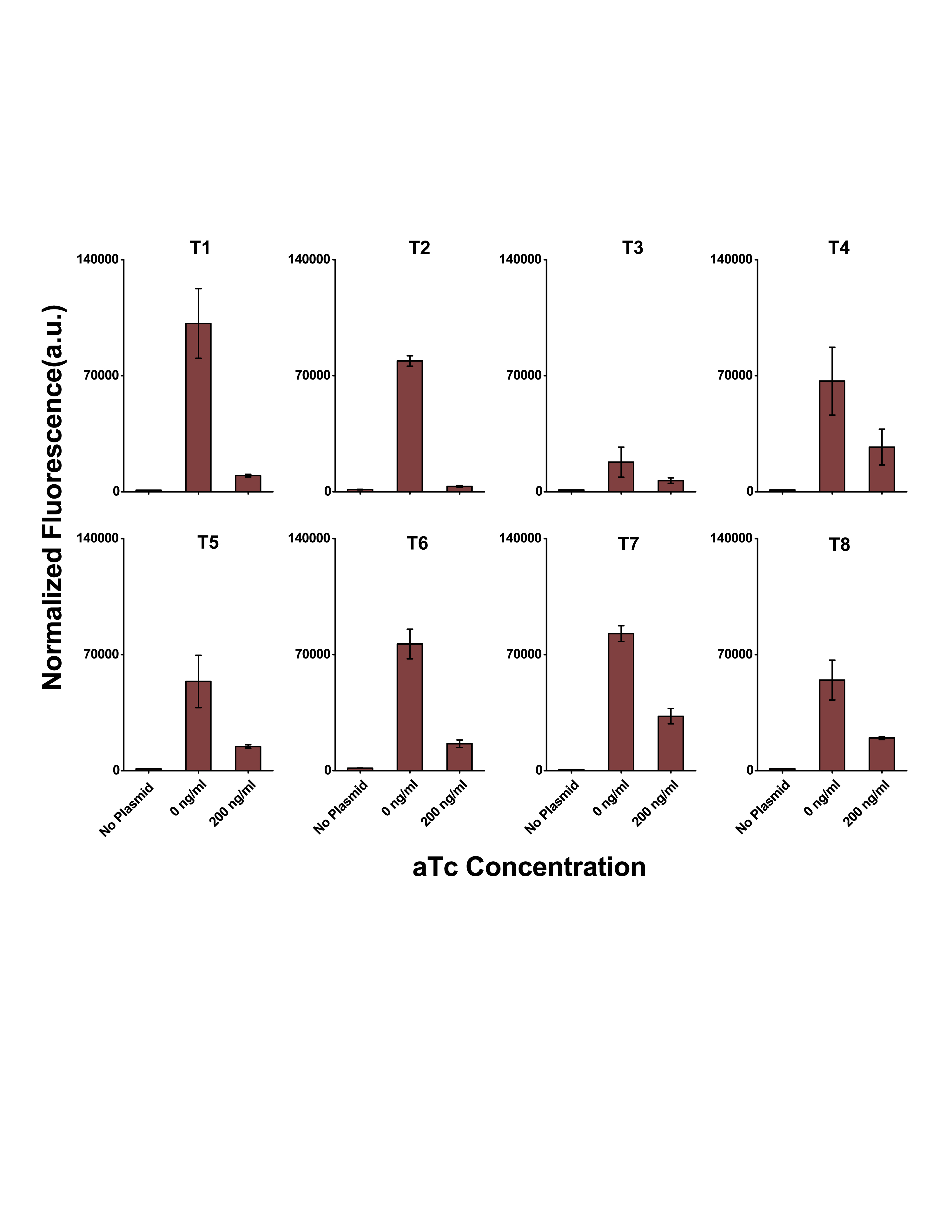


**Figure S6. Characterization of the NOT gates repression behaviour with postulated truncated variants of cI.** Normalized EGFP fluorescence for the NOT gate constructs T1-T8 is measured at 0 ng/ml and 200 ng/ml of aTc and compared to the autofluorescence coming from the DH5αZ1 cells carrying no plasmid (no plasmid panel).

**Table S1**- **Curve fitting parameter values.** aTc dose response curves for synthetic NOT gates Fs1, Fs2, Fs4, Fs5 and Fs7 are fitted with Equation (1) (main text) into OriginLab and parameter values are extracted.

| **Construct name** | **Parameter Values with standard error** | | | | | | | |
| --- | --- | --- | --- | --- | --- | --- | --- | --- |
|  | **c** | | **b** | | **K** | | **n** | |
|  | **Value** | **Standard error** | **Value** | **Standard error** | **Value** | **Standard error** | **Value** | **Standard error** |
| Fs1 | 0.86455 | 0.03328 | 0.00486 | 3.79293×10^-4^ | 8.54199 | 0.13548 | 6.64304 | 0.65016 |
| Fs2 | 0.84768 | 0.02594 | 0.00301 | 4.24886×10^-4^ | 5.69962 | 0.368 | 3.63556 | 0.30411 |
| Fs4 | 0.81502 | 0.00919 | 0.00602 | 2.65363×10^-4^ | 9.83616 | 0.46925 | 4.17457 | 0.3828 |
| Fs5 | 0.70768 | 0.02194 | -1.97397× 10^-4^ | 4.97192×10^-4^ | 7.56069 | 0.51722 | 5.62459 | 1.21501 |
| Fs7 | 0.83055 | 0.04969 | 0.02755 | 0.00237 | 11.95655 | 1.13367 | 5.03001 | 1.02773 |

**Table S2- Translation initiation rates for EGFP and cI calculated from RBS calculator.**

| **Name of the gene** | **Promoter regulating the gene expression** | **Translation initiation rate (a.u.)** |
| --- | --- | --- |
| *EGFP* | P_LtetO-1_ | 34.81 |
| *cI* | P_LtetO-1_ | 40768.91 |

**Table S3- List of promoters**. Restriction enzyme sites are marked in italics. *tet*O2, *lac*O1, *O*_R_1 and *O*_R_2 sites are colored in blue, green, pink and orange respectively. RBS is colored in red. Transcription start site is shown in bold. -10 and -35 hexamers are underlined.

| **Promoter name** | **Sequence(5'- 3')** |
| --- | --- |
| P_LtetO-1_ | *CTCGAG*TCCCTATCAGTGATAGAGATTGACATCCCTATCAGTGATAGAGATACTGAGCAC**A**TCAGCAGGACGCACTGACC*GAATTC*ATTAAAGAGGAGAAA*GGTACC* |
| P_LlacO-1_ | *CTCGAG*AATTGTGAGCGGATAACAATTGACATTGTGAGCGGATAACAAGATACTGAGCAC**A**TCAGCAGGACGCACTGACC*GAATTC*ATTAAAGAGGAGAAA*GGTACC* |
| P_R_ | *CTCGAG*TAACACCGTGCGTGTTGACTATTTTACCTCTGGCGGTGATAATGGTTGC**A**TGTAC*GAATTC*ATTAAAGAGGAGAAA*GGTACC* |
| P_LTATAA_ | *CTCGAG*TCCCTATCAGTGATAGAGATTGACATTGTGAGCGGATAACAAGATACTGAGCAC**A**TCCCTATCAGTGATAGAGAGATAATTGTGAGCGGATAACAATTGATAATTGTGAGCGGATAACAATT*GAATTC*ATTAAAGAGGAGAAA*GGTACC* |

**Table S4- List of plasmids**. Ampicillin, chloramphenicol and kanamycin are abbreviated as Amp, Cm and Kan respectively.

| **Plasmid Name** | **Gene of interest** | **Promoter** | **Origin of replication** | **Antibiotic selection** | **Source** |
| --- | --- | --- | --- | --- | --- |
| pOR-EGFP-12 | *EGFP* | P_LlacO-1_ | ColE1 | Amp | Prof. David McMillen |
| pOR-Luc-31 | *Luc* | P_LtetO-1_ | P15A | Cm | Prof. David McMillen |
| pmCherry-N1 | *mCherry* | - | pUC | Kan | Clontech |
| pTA1EGFP | *EGFP* | P_LtetO-1_ | pUC | Amp | This study |
| pTA2EGFP | *EGFP* | P_LtetO-1_ | ColE1 | Amp | This study |
| pTC3EGFP | *EGFP* | P_LtetO-1_ | P15A | Cm | This study |
| pLA1SEGFP | *EGFP* | P_LlacO-1_ | pUC | Amp | This study |
| pRA1EGFP | *EGFP* | P_R_ | pUC | Amp | This study |
| pRA2EGFP | *EGFP* | P_R_ | ColE1 | Amp | This study |
| pRC3EGFP | *EGFP* | P_R_ | P15A | Cm | This study |
| pRA1SEGFP | *EGFP* | P_R_ | pUC | Amp | This study |
| PRA2SEGFP | *EGFP* | P_R_ | ColE1 | Amp | This study |
| PRA3SEGFP | *EGFP* | P_R_ | P15A | Amp | This study |
| pDA1EGFP | *EGFP* | P_LTATAA_ | pUC | Amp | This study |
| pDA1SEGFP | *EGFP* | P_LTATAA_ | pUC | Amp | This study |
| pDA2EGFP | *EGFP* | P_LTATAA_ | ColE1 | Amp | This study |
| pTA1cI | Wild type *cI* | P_LtetO-1_ | pUC | Amp | This study |
| pTA2cI | Wild type *cI* | P_LtetO-1_ | ColE1 | Amp | This study |
| pTC3cI | Wild type *cI* | P_LtetO-1_ | P15A | Cm | This study |
| pRA1SEGFPTcI | *EGFP* and wild type *cI* | *EGFP* under P_R_ and wild type *cI* under P_LtetO-1_ | pUC | Amp | This study |
| pRA2SEGFPTcI | *EGFP* and wild type *cI* | *EGFP* under P_R_ and wild type *cI* under P_LtetO-1_ | ColE1 | Amp | This study |
| pRC3SEGFPTcI | *EGFP* and wild type *cI* | *EGFP* under P_R_ and wild type *cI* under P_LtetO-1_ | P15A | Cm | This study |
| pTA1cIfm | Frame-shifted *cI* | P_LtetO-1_ | pUC | Amp | This study |
| pTA2cIfm | Frame-shifted *cI* | P_LtetO-1_ | ColE1 | Amp | This study |
| pTc3cIfm | Frame-shifted *cI* | P_LtetO-1_ | P15A | Cm | This study |
| PLA1ScIfm | Frame-shifted *cI* | P_LlacO-1_ | pUC | Amp | This study |
| pLA2ScIfm | Frame-shifted *cI* | P_LlacO-1_ | ColE1 | Amp | This study |
| pRA1SEGFPTcIfm | *EGFP* and frame-shifted *cI* | *EGFP* under P_R_ and frame-shifted *cI* under P_LtetO-1_ | pUC | Amp | This study |
| pRA2SEGFPTcIfm | *EGFP* and frame-shifted *cI* | *EGFP* under P_R_ and frame-shifted *cI* under P_LtetO-1_ | ColE1 | Amp | This study |
| pRC3SEGFPTcIfm | *EGFP* and frameshifted *cI* | *EGFP* under P_R_ and frameshifted *cI* under P_LtetO-1_ | P15A | Cm | This study |
| pRA3SEGFPTcIfm | *EGFP* and frame-shifted *cI* | *EGFP* under P_R_ and frame-shifted *cI* under P_LtetO-1_ | P15A | Amp | This study |
| pLA2ScIfmTcIfm | Two copies of  frame-shifted *cI* | Frame-shifted *cI* under both P_LlacO-1_ and P_LtetO-1_ | ColE1 | Amp | This study |
| pDA2ScIfm | Frame-shifted *cI* | P_LTATAA_ | ColE1 | Amp | This study |
| pTA1cI-G | *cI*-G | P_LtetO-1_ | pUC | Amp | This study |
| pTA1cI-T3 | *cI*-T3 | P_LtetO-1_ | pUC | Amp | This study |
| pTA1cI-K4 | *cI*-K4 | P_LtetO-1_ | pUC | Amp | This study |
| pTA1cI-K5 | *cI*-K5 | P_LtetO-1_ | pUC | Amp | This study |
| pTA1cI-K6 | *cI*-K6 | P_LtetO-1_ | pUC | Amp | This study |
| pTA1cI-L8 | *cI*-L8 | P_LtetO-1_ | pUC | Amp | This study |
| pTA1cI-T9 | *cI*-T9 | P_LtetO-1_ | pUC | Amp | This study |
| pTA1cI-E11 | *cI*-E11 | P_LtetO-1_ | pUC | Amp | This study |
| pTA1cI-M41 | *cI*-M41 | P_LtetO-1_ | pUC | Amp | This study |

**Table S5- List of primers**. All primers are synthesized from Integrated DNA Technologies Pvt Ltd.

| **Primer name** | **Sequence (5' - 3')** | **Purpose** |
| --- | --- | --- |
| Primer_1 | AAAGAAACCATTAACACAAGAGCA | 1^st^ round amplification of wt *cI*, *cI*-G and *cI*-T3 (Forward primer) |
| Primer_2 | TCAGCCAAACGTCTCTTCAG | 1^st^ round amplification of wt *cI, cI-*G and *cI*-T3 (Reverse primer) |
| Primer_3 | GTCCAGTTGAGGTACCATGAGCACAAAAAAGAAACCATTAACACAAGAGCA | 2^nd^ round amplification of wt *cI* (Forward primer) |
| Primer_4 | GAGCATATCGTCTAGATCAGCCAAACGTCTCTTCAG | 2^nd^ round amplification of wt *cI, cI*-G and *cI*-T3 (Reverse primer) |
| Primer_5 | CAAGGGCGAGGAGCTGTT | 1^st^ round amplification of *EGFP* (Forward primer) |
| Primer_6 | CCATGCCGAGAGTGATCC | 1^st^ round amplification of *EGFP* (Reverse primer) |
| Primer_7 | CTTCAGTCGAGGTACCATGGTGAGCAAGGGCGAGGAGCTGTT | 2^nd^ round amplification of *EGFP* (Forward primer) |
| Primer_8 | CTGATTATGATCTAGATTACTTGTACAGCTCGTCCATGCCGAGAGTGATCC | 2^nd^ round amplification of *EGFP* (Reverse primer) |
| Primer_9 | TCGTCTTCGGCTCGAGTAACACCGTGCGTGTTGACTATTTTACCTCTGGCGGTGATAATGGTT | 1^st^ round amplification of P_R_ promoter (Forward primer) |
| Primer_10 | TGAACAGCTCCTCGCCCTTGCTCACCATGGTACCTTTCTCCTCTTTAATGAATTCGTACATGCAACCATTATCACCGCCAGAG | 1^st^ round amplification of P_R_ promoter (Reverse primer) |
| Primer_11 | TCGTCTTCGGCTCGAGTAAC | 2^nd^ round amplification of P_R_ promoter (Forward primer) |
| Primer_12 | GCCCTTTCGTCTTCACCTC | Amplification of P_LTATAA_ promoter (Forward primer) |
| Primer_13 | CTTGACTGGAATTCAATTGTTATCCGCTCACAATTATCAATTGTTATCCGCTCACAATTATCTCTCTATCACTGATAGGGATGTGCTCAG | Amplification of P_LTATAA_ promoter (Reverse primer) |
| Primer_14 | CTGATTATGACTCGAGAATTGTGAGCGGATAACAATTGACATTGTGAGCGGATAACAAGATACTGAGC | Amplification of P_LlacO-1_ promoter (Forward primer) |
| Primer_15 | CTCCAGTCGTGAATTCGGTCAGTGCGTCCTGCTGATGTGCTCAGTATCTTGTTATCCGCTC | Amplification of P_LlacO-1_promoter (Reverse primer) |
| Primer_16 | GTCCAGTTGAGGTACCGGCACAAAAAAGAAACCATTAACACAAGAGCA | 2^nd^ round amplification of *cI*-G (Forward primer) |
| Primer_17 | GTCCAGTTGAGGTACCACAAAAAAGAAACCATTAACACAAGAGCA | 2^nd^ round amplification of *cI*-T3 (Forward primer) |
| Primer_18 | ACACAAGAGCAGCTTGAGGAC | 1^st^ round amplification of *cI*-K4, *cI*-K5, *cI*-K6, *cI*-L8 and *cI*-T9 (Forward primer) |
| Primer_19 | AGCGATAACTTTCCCCACAA | 1^st^ round amplification of *cI*-K4, *cI*-K5, *cI*-K6, *cI*-L8 and *cI*-T9 (Reverse primer) |
| Primer_20 | GTCCAGTTGAGGTACCAAAAAGAAACCATTAACACAAGAGCA | 2^nd^ round amplification of *cI*-K4 (Forward primer) |
| Primer_21 | GACCAGTTGAGGTACCAAGAAACCATTAACACAAGAGCAGCTTGAGGAC | 2^nd^ round amplification of *cI*-K5 (Forward primer) |
| Primer_22 | GACCAGTTGAGGTACCAAACCATTAACACAAGAGCAGCTTGAGGAC | 2^nd^ round amplification of *cI*-K6 (Forward primer) |
| Primer_23 | GACCAGTTGAGGTACCTTAACACAAGAGCAGCTTGAGGAC | 2^nd^ round amplification of *cI*-L8 (Forward primer) |
| Primer_24 | GACCAGTTGAGGTACCACACAAGAGCAGCTTGAGGAC | 2^nd^ round amplification of *cI*-T9 (Forward primer) |
| Primer_25 | GACCATGTCGTCTAGATCAGCCAAACGTCTCTTCAGGCCACTGACTAGCGATAACTTTCCCCACAA | 2^nd^ round amplification of *cI*-K4, *cI*-K5, *cI*-K6, *cI*-L8 and *cI*-T9 (Reverse primer) |
| Primer_26 | GAACTTGGCTTATCCCAGGAA | 1^st^ round amplification of *cI*-E11 (Forward primer) |
| Primer_27 | AGCGATAACTTTCCCCACAAC | 1^st^ round amplification of *cI*-E11 (Reverse primer) |
| Primer_28 | CCAGATGAGGTACCGAGCAGCTTGAGGACGCACGTCGCCTTAAAGCAATTTATGAAAAAAAGAAAAATGAACTTGGCTTATCCCAGGAA | 2^nd^ round amplification of *cI*-E11 (Forward primer) |
| Primer_29 | GACCATGTCGTCTAGATCAGCCAAACGTCTCTTCAGGCCACTGACTAGCGATAACTTTCCCCACAAC | 2^nd^ round amplification of *cI*-E11 (Reverse primer) |
| Primer_30 | GTCAGGCGTTGGTGCTTTAT | 1^st^ round amplification of *cI*-M41 (Forward primer) |
| Primer_31 | AGCGATAACTTTCCCCACAAC | 1^st^ round amplification of *cI*-M41 (Reverse primer) |
| Primer_32 | GTCCGATTGAGGTACCATGGGGATGGGGCAGTCAGGCGTTGGTGCTTTAT | 2^nd^ round amplification of *cI*-M41 (Forward primer) |
| Primer_33 | GAACATAGCGTCTAGATCAGCCAAACGTCTCTTCAGGCCACTGACTAGCGATAACTTTCCCCACAAC | 2^nd^ round amplification of *cI*-M41 (Reverse primer) |
| Primer_34 | GCAGGAAAGACCTAGGGGCTGGCACTCTGTCGATAC | Amplification of pUC ori (Forward primer) |
| Primer_35 | GACTCCAAGCACTAGTAGGGGATAACGCAGGAAAGA | Amplification of pUC ori (Reverse primer) |
| Primer_36 | CTGTTCATATCTGCAGTTCGTCTTCACCTCGAGTCC | Incorporation of *Pst*I site to the gene cassette having P_LtetO-1_ promoter |
| Primer_37 | CTCCAGTCGTGGATCCATTACCGCCTTTGAGTGAGC | Incorporation of *Bam*HI site to the gene cassette having ColE1origin |
| Primer_38 | CTGTTCATATCTGCAGCGTATCACGAGGCCCTTTC | Incorporation of *Pst*I site to the gene cassette having any promoter |
| Primer_39 | CTCCAGTCGTGGATCCCGGCGGATTTGTCCTACTC | Incorporation of *Bam*HI site to the gene cassette having any origin |
| Primer_40 | GCTTTTGGCGAAGAATGAAA | Sequencing of the gene cassette flanked by *Bam*HI and *Pst*I (Forward primer with respect to the promoter) |
| Primer_41 | GTTTTGGAGCACGGAAAGAC | Sequencing of the gene cassette flanked by *Bam*HI and *Pst*I (Reverse primer with respect to the promoter) |
| Primer_42 | CGAAAAGTGCCACCTGAC | Sequencing of the vectors having P_LtetO-1_ and P_LTATAA_ promoters |

References:

1. Bagh S, Mazumder M, Velauthapillai T, Sardana V, Dong GQ, Movva AB, et al. Plasmid-borne prokaryotic gene expression: sources of variability and quantitative system characterization. Phys Rev E.2008;77: 021919.
2. Lutz R, Bujard H. Independent and tight regulation of transcriptional units in Escherichia coli via the LacR/O, the TetR/O and AraC/I1-I2 regulatory elements. Nucleic Acids Res.1997;25: 1203-10.
